# PD-L1 expression comparison between primary and relapsed non-small cell lung carcinoma using whole sections and clone SP263

**DOI:** 10.18632/oncotarget.25770

**Published:** 2018-07-13

**Authors:** Enrico Munari, Giuseppe Zamboni, Gianluigi Lunardi, Marcella Marconi, Marco Sommaggio, Matteo Brunelli, Guido Martignoni, George J. Netto, Mohammad O. Hoque, Francesca Moretta, Maria Cristina Mingari, Maria Cristina Pegoraro, Francesca Romana Mariotti, Paola Vacca, Lorenzo Moretta, Giuseppe Bogina

**Affiliations:** ^1^ Department of Pathology, Sacro Cuore Don Calabria Hospital, 37024 Negrar VR, Italy; ^2^ Department of Diagnostics and Public Health, University of Verona, 37134 Verona VR, Italy; ^3^ Department of Oncology, Sacro Cuore Don Calabria Hospital, 37024 Negrar VR, Italy; ^4^ Department of Pathology, Pederzoli Hospital, 37019 Peschiera del Garda VR, Italy; ^5^ Department of Pathology, The University of Alabama at Birmingham, Birmingham, AL 35233, USA; ^6^ Department of Otolaringology, Johns Hopkins University School of Medicine, Baltimore, MD 21287, USA; ^7^ Department of Laboratory Medicine, Sacro Cuore Don Calabria Hospital, 37024 Negrar VR, Italy; ^8^ Department of Experimental Medicine (DIMES), University of Genova, 16132 Genova GE, Italy; ^9^ Department of Oncology, Pederzoli Hospital, 37019 Peschiera del Garda VR, Italy; ^10^ Immunology Research Area, IRCCS Bambino Gesù Pediatric Hospital, 00146 Rome RM, Italy

**Keywords:** PD-L1, lung, cancer, heterogeneity, metastases

## Abstract

We assessed the concordance, in terms of PD-L1 expression, between primary and metastatic non-small cell lung carcinoma (NSCLC) of different histotypes using validated SP263 clone. A few samples of local recurrences have also been analyzed.

Whole sections of consecutive cases of primary NSCLC and paired relapses undergone surgical resection have been stained with PD-L1 clone SP263; for scoring purposes, a three-tiered system was applied using the following thresholds: <1%, 1–49% and ≥50%.

Eighty-four cases of paired primary and relapsed tumors from 83 patients were analyzed, including 75 metastases and 9 local recurrences. Regarding metastases, when considering a cutoff of 1%, discrepancy in PD-L1 expression occurred in 9/75 (12%) paired samples (kappa value = 0.75); at 50% cutoff, discrepancy in PD-L1 expression was detected in 7/75 (9.3%) of paired samples (kappa value = 0.61). Regarding recurrences, at 1% cutoff, the discrepancy in PD-L1 expression was seen in 3/9 (33%) paired samples and in all cases there was a gained PD-L1 expression; at 50% cutoff, 1/9 (11%) paired samples showed gained PD-L1 expression.

Our data provide important information regarding the concordance between primary and relapsed NSCLC and the degree of reliability of metastatic sites in terms of PD-L1 expression evaluation.

## INTRODUCTION

Programmed cell death 1 (PD1) is an inhibitory receptor originally identified in T lymphocytes and, more recently, in NK cells, that, upon interaction with its ligand PD-L1, delivers inhibitory signals resulting in downregulation of T cell function. Under physiological conditions, this interaction represents an important checkpoint in the immune responses leading to peripheral T-cell tolerance. On the other hand, cancer cells can acquire the ability to exploit such interaction to evade immune surveillance by de novo acquiring PD-L1 and, possibly, by favoring the expression of PD1 on T and NK cells [1–3].

This process provided the rationale for a new approach of immunotherapy based on the use of checkpoint inhibitors, i.e. blocking monoclonal antibodies specific for PD1 or PDL1. This approach has proven highly effective in different tumor types, thus representing a major turning point in cancer therapy [4–6].

Currently, there are four drugs targeting the PD1/PD-L1 axis which have been approved by the Food and Drug Administration (FDA): two are directed to PD-L1 (atezolizumab and durvalumab) and two are specific for PD1 (nivolumab and pembrolizumab). Nivolumab was initially approved for patients with non-small cell lung cancer (NSCLC) with squamous histology and subsequently extended to patients with non-squamous NSCLC [7]. Pembrolizumab is an anti-PD1 humanized monoclonal antibody that has recently been granted FDA approval after clinical trials in patients with advanced lung adenocarcinoma or squamous carcinoma that expressed PD-L1 on viable tumor cells, evaluated with a validated assay. Specifically, pembrolizumab has been shown to improve the overall survival in previously treated patients whose tumors expressed PD-L1 in at least 1% of cells [8]. Importantly, the results of another trial revealed significantly longer progression-free survival and overall survival for previously untreated patients with tumors expressing PD-L1 in at least 50% of cells [9, 10]. In view of these results, the immunohistochemical evaluation of PD-L1 expression on tumor specimens has become an issue of major diagnostic and prognostic value.

In many cases, such evaluation is made on metastatic lesions through biopsy sampling. However, it is of note that PD-L1 expression may be rather heterogeneous within a primary NSCLC; in this regard, we have recently shown that when only one random biopsy is available, a significant proportion of cases may be misclassified [11]. In addition, since PD-L1 expression on neoplastic cells is regulated by different mechanisms that may occur during the metastatic process and/or may be induced by the environmental conditions present at different metastatic sites [12], it is possible that relevant discrepancies between primary and metastatic tumors may actually exist. In order to investigate this issue, the present study was designed to assess the concordance, in terms of PD-L1 expression, between primary and metastatic NSCLCs of different histotypes using the validated SP263 clone. A few samples of local recurrences have also been analyzed.

## RESULTS

### Patient characteristics

From an initial cohort of 271 consecutive patients, we retrieved 84 cases of paired primary and relapsed tumors from 83 patients: 75 metastases (71 to tumor-draining lymph nodes and 4 to distant sites) and 9 local recurrences. All cases were retrieved from a single institution (Sacro Cuore Don Calabria Hospital); none received systemic therapy or radiation prior tumor resection/biopsy.

Clinical and pathological features of corresponding primary tumors are shown in Table 1.

**Table 1:** Clinical and pathological features of primary tumors

Patients	83
**Age**	
<70 y	40
≥70 y	43
**Sex**	
Male	68
Female	15
**Histology**	
ADC	58
SCC	16
yOthers	9
**Diameter**	
y≤30 mm	48
>30 mm	35
**N Stage**	
N0	10
N1	34
N2	35
N3	4

ADC: adenocarcinoma; SCC: squamous cell carcinoma.

All lymph node metastases were synchronous (33 N1, 34 N2 and 4 N3), while all distant metastases were metachronous, with a mean interval from time of resection of the primary tumor of 19 months (range: 8–29 months); sites of distant metastases were: colon (1), skin (1), contralateral clavicular node (1) and contralateral lung (1).

Mean time interval to local recurrence was 40 months (range 8–91 months).

All but one samples analyzed were surgical specimens.

### PD-L1 expression status in primary tumors and paired metastatic lesions

PD-L1 expression status in primary tumors and paired metastases are shown in Table 2.

**Table 2:** PD-L1 expression status in primary tumors and paired metastases

Primary		Metastases		
	**<1%**	**1–49%**	**≥50%**	**Total**
**<1%**	40	2	0	42
**1–49%**	5	14	2	21
**≥50%**	2	3	7	12
**Total**	47	19	9	75

When considering a cutoff of 1%, discrepancy in PD-L1 expression occurred in 9/75 (12%) paired samples (kappa value = 0.75): in 7 (9.3%) cases PD-L1 expression in the metastasis was lost while in 2 cases (2.7%) was gained.

When considering a cutoff of 50%, discrepancy in PD-L1 expression was detected in 7/75 (9.3%) of paired samples (kappa value = 0.61): in 5 cases (6.6%) expression was lost in the metastasis while in 2 cases (2.7%) it was gained.

All discordant paired specimens of metastases were in tumor draining lymph nodes. Indeed, all cases of distant metastases and the corresponding primary tumors resulted negative for PD-L1.

**Figure 1:**
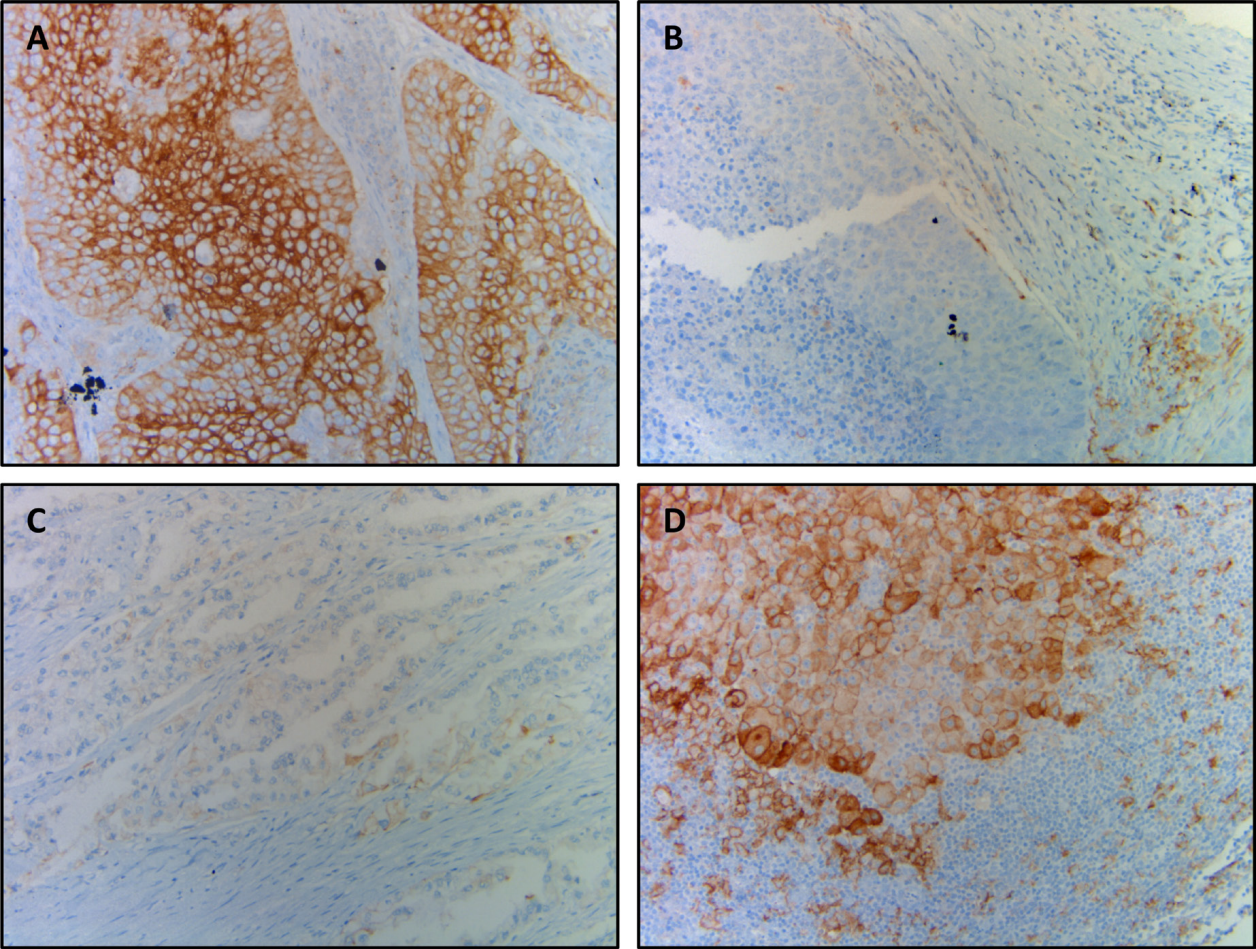
Representative photographs of discrepant PD-L1 expression between primary and metastatic tumors (**A** and **B**) Squamous cell carcinoma showing strong and diffuse PD-L1 positivity in ≥50% of cells (A) and corresponding nodal metastasis showing no PD-L1 staining (B); (**C** and **D**) adenocarcinoma showing focal and dim PD-L1 expression in <50% of neoplastic cells (C) and corresponding nodal metastasis showing diffuse PD-L1 expression in ≥50% of cells (D).

Figure 1 shows representative images of a primary and paired metastatic tumor with discrepancy in PD-L1 expression.

### PD-L1 expression status in primary tumors and paired local recurrences

PD-L1 expression status in primary tumors and paired local recurrences are shown in Table 3.

**Table 3:** PD-L1 expression status in primary tumors and paired local recurrences

Primary		Recurrences		
	**<1%**	**1–49%**	**≥50%**	**Total**
**<1%**	5	2	1	8
**1–49%**	0	1	0	1
**≥50%**	0	0	0	0
**Total**	5	3	1	9

It can be seen that at 1% cutoff, the discrepancy in PD-L1 expression was seen in 3/9 (33%) paired samples and in all cases there was a gained PD-L1 expression; at 50% cutoff, 1/9 (11%) paired samples showed gained PD-L1 expression in the recurrent tumor.

## DISCUSSION

Immunotherapy with inhibitors of PD1/PD-L1 axis represents a true revolution in oncology and a starting point for a whole new therapeutic strategy against cancer [13].

Identification of those patients who will respond to checkpoint inhibitors therapy is a major issue that so far has been based mainly on immunohistochemical evaluation of PD-L1 expression on tumor cells. In this context, although different studies revealed a significant correlation between expression of PD-L1 and response to checkpoint inhibitors in different tumor types, in some reports response to therapy has been described also in patients whose tumors lacked PD-L1 expression [6, 9, 10, 14–17]. In any case, therapy with Nivolumab was found to be associated with prolonged survival in patients with non-squamous NSCLC expressing higher levels of PD-L1 on neoplastic cells [16].

Notably, the FDA recently approved the anti-PD1 pembrolizumab as a single agent for patients with tumors expressing PD-L1 in ≥50% of neoplastic cells as first line therapy and as a second line therapy for patients whose tumors express PD-L1 in at least 1% of cells [9, 10]. Therefore, evaluation of PD-L1 expression by immunohistochemistry on tumor specimens is playing a critical role in the selection of patients who could benefit from therapy with PD1/PDL1-specific checkpoint inhibitors. In this context, it is important to underline that the majority of these patients have advanced tumors and that, in most instances, tissues to be analyzed are obtained from metastatic sites. Thus, information on PD-L1 expression in metastatic lesions and the degree of concordance with primary tumors is particularly relevant. In addition, since the heterogeneity of PD-L1 expression represents an important issue for a correct quantification assessment in primary tumors, another problem to be considered is the possible discrepancy in PD-L1 expression in primary tumors and in paired relapses. In this regard, very few studies have been performed.

Kim MY *et al*. compared PD-L1 (clone E1L3N, Cell Signaling) expression between primary and nodal metastases in 77 cases of lung squamous cell carcinomas and found discrepancy in 30% of the overall cases [18].

Uruga *et al*. compared the PD-L1 (clone E1L3N, Cell Signaling) expression in primary tumor and metastatic lymph nodes in 66 cases of lung adenocarcinoma and found that up to 38% of cases showed discrepant PD-L1 expression between primary tumor and paired metastatic lymph nodes, raising the possibility of an heterogeneous intertumoral PD-L1 expression; however, after dichotomizing cutoffs at 1% and 50% of cells, PD-L1 resulted to be concordant respectively in 74% and 88% of cases [19]. Kim S *et al*. analyzed PD-L1 (clone E1L3N, Cell Signaling) expression between primary lung adenocarcinomas and paired nodal lymph node metastases in a cohort of 161 patients and found an overall concordance rate of 75.2% while, using 1% and 50% cutoffs, the reported concordance rate was 80% and 90,7% of cases, respectively [20]. Notably, compared with the latter study, our data show a higher concordance rate at 1% between primary and metastatic NSCLC (88% of cases). In our present study, we also analyzed PD-L1 expression in primary NSCLC and local recurrences in 9 cases. Discrepancy was found in 33% and 11% at 1% and 50% cutoff, respectively. Despite the limited number of cases analyzed, data suggest that locally relapsed tumors may display a different profile in terms of PD-L1 expression, possibly reflecting more complex and heterogeneous biological properties of neoplastic cells as compared to primary tumors.

At variance with the aforementioned reports, in the present study, we analyzed NSCLC belonging to different histotypes, including adenocarcinomas, squamous cell carcinomas and large cell carcinomas; moreover, a validated antibody was used to assess PD-L1 expression (Ventana's SP263).

To our knowledge, no prior study assessed PD-L1 expression between primary tumors and paired relapses using a validated immunohistochemical assay.

In fact, the availability of four different approved PD-L1 IHC assays poses problems for the application of PD-L1 testing regarding which antibody and platform should be used. Harmonization studies have conducted with conflicting results: while some studies indicated that 3 clones, specifically 22C3 (Dako), 28–8 (Dako) and SP263 (Ventana) are comparable [21, 22], other studies, including ours, have reported that these assays are not interchangeable [23, 24]. Since the immunohistochemistry platform available in our institution is Ventana, we used clone SP263 for the evaluation and scoring of our specimens. Moreover, Ventana's SP263 is CE-marked to inform treatment decisions for nivolumab and pembrolizumab in NSCLC.

Importantly, a recent harmonization study found that almost half of the laboratory developed tests (LDT) did not achieve a sufficient correlation for tumor cells staining with the 3 reference PD-L1 assays (28–8, 22C3, SP263). Specifically, regarding clone E1L3N, it failed to reach a sufficient weighted kappa concordance coefficient (≥ 0.75) in 3 out of 7 centers using three platforms (Dako, Ventana and Leica), considering SP263 as reference [25].

In another study, the SP263 IHC assay has been deemed superior to the E1L3N IHC assay due to its staining intensity, scoring range and pathologist preference [26].

The fact that a proportion of patients respond to anti PD1/PD-L1 antibodies even though their tumors do not express PD-L1 could be, at least in part, explained by the intratumoral and intertumoral heterogeneity of PD-L1. In this regard, we recently proposed a method for harmonization of PD-L1 expression between core biopsies and whole tumor sections for better stratification of patients [27]. In addition, according to our present data, it is clear that some cases may be misclassified due to the discrepant expression of PD-L1 between primary and metastatic tumors. It is conceivable that, in view of the complex mechanisms of immune response against tumors, other molecules may be involved in the inhibitory interactions. In fact, PD-L2 expression has been shown to be predictive of longer progression free survival in patients with head and neck squamous cell carcinoma treated with pembrolizumab (anti-PD1) [28]. In this context, it is reasonable to that, in the future, it will be crucial to evaluate the status of other molecules, including PD-L2, in order to better stratify the patients eligible for treatment with checkpoint inhibitors.

In conclusion, we found PD-L1 expression concordance between primary and paired metastatic tumor in 88% and 90.7% of cases using 1% and 50% cutoff, respectively; regarding local tumor recurrences, a lower concordance (66%) at 1% cutoff was detected, suggesting the possible need for tumor re-biopsy in the setting of second line pembrolizumab treatment. Overall, our data provide important information regarding the concordance between primary and relapsed NSCLC and the degree of reliability of metastatic sites in terms of PD-L1 expression evaluation.

However, further studies including new molecules involved in the interplay between cancer and immune cells are needed in order to further refine our ability to select those patients who are likely to benefit from immunotherapy with checkpoint inhibitors.

## MATERIALS AND METHODS

### Study cohort

The study cohort consisted of consecutive patients with primary NSCLC and paired relapses who had undergone surgical resection at the Sacro Cuore Don Calabria Hospital of Negrar, Verona (Italy) between 2003 and 2017 with available slides and paraffin embedded tissue blocks. None of the patients received therapy before surgery.

Tumors were classified according to the 2015 WHO classification [29]. Staging was performed using the TNM staging manual (7th edition). Patients demographics and clinical data were retrieved from the digital archives.

Investigations have been conducted according to principles expressed in the Declaration of Helsinki.

### Immunohistochemistry and scoring

From each block 5 μm sections were cut and stained with anti-PD-L1 (clone SP263, Ventana) on an automated staining platform (Benchmark ULTRA; Ventana). An OptiView DAB IHC Detection Kit (Ventana) and an OptiView Amplification Kit (Ventana) were used according to the manifacturer's recommendations for the visualization of the primary anti PD-L1 antibody.

Stained sections were scanned using Ventana iScan HT and scored based on the percentage of positive tumor cells, irrespective of the staining intensities; a three-tiered system was then applied using the following thresholds: <1%, 1–49% and ≥50%.

Macrophages were used as internal control in order to validate the adequacy of PD-L1 staining reaction.

### Statistical analysis

Statistical analysis was carried out using Stata; Cohen's κ was used to calculate coefficient of agreement.
